# COMMUNITY TYPOLOGY IN OLDER KOREAN AMERICANS: IMPLICATIONS FOR MENTAL/COGNITIVE HEALTH

**DOI:** 10.1093/geroni/igac059.2300

**Published:** 2022-12-20

**Authors:** Nan Sook Park, Yuri Jang, Soondool Chung, David Chiriboga

**Affiliations:** University of South Florida, Tampa, Florida, United States; University of Southern California, Los Angeles, California, United States; Ewha Womans University, Seoul, Seoul-t'ukpyolsi, Republic of Korea; University of South Florida, Tampa, Florida, United States

## Abstract

The purposes of this study were to: (1) identify community typology in older Korean Americans; and (2) examine the associations of typology with loneliness, mental distress, and cognitive health. Guided by social capital conceptualization, we hypothesized that distinct community groups would be identified and that they would be differentially associated with sociodemographic, immigration-related, health, and social characteristics and mental/cognitive health. Data were drawn from a survey with older Korean Americans aged 60 and older, collected during 2017−2018 in diverse locations (n=2,138). To identify community typology, a series of latent profile analysis (LPA) were conducted using 15 community-related variables in three domains (neighborhood characteristics, social cohesion, ethnic attachment). After examining characteristics of the identified groups in relations with study variables, hierarchical multiple regression models of loneliness, mental distress, and self-rated cognitive health were estimated. Based on several model evaluation criteria, LPA model with five community groups was identified as best-fit (BIC=64,619, Entropy=.94). The five groups were identified as “a. high safety/cohesion/ethnic attachment” (10%), “b. high safety/low cohesion/ethnic attachment” (10%), “c. moderate neighborhood/low ethnic attachment/cohesion” (38%), “d. moderate neighborhood/high cohesion/ethnic attachment” (31%), and “e. low safety/moderate cohesion/ethnic attachment” (11%). In reference to the group with high on all three domains (a), group with low ethnic attachment/cohesion in moderate neighborhood (c) and group in unsafe environment with moderate cohesion/ethnic attachment (e) were consistently associated with elevated loneliness/mental distress and poor rating of cognitive health. The results suggest the need to understand profiles of community characteristics and their relationships with health/well-being among older immigrants.

